# *Clostridium perfringens* sialidase interaction with Neu5Ac α-Gal sialic acid receptors by *in-silico* observation and its impact on monolayers cellular behavior structure

**DOI:** 10.5455/javar.2023.j722

**Published:** 2023-12-31

**Authors:** Ryan Septa Kurnia, Amin Soebandrio, Vivi Hardianty Harun, Christian Marco Hadi Nugroho, Desak Gede Budi Krisnamurti, Okti Nadia Poetri, Agustin Indrawati, Simson Tarigan, Ketut Karuni Nyanakumari Natih, Fera Ibrahim, Pratiwi Pudjilestari Sudarmono, Otto Sahat Martua Silaen

**Affiliations:** 1 Doctoral Program in Biomedical Science, Faculty of Medicine, Universitas Indonesia, Jakarta, Indonesia; 2 Department of Microbiology, Faculty of Medicine, Universitas Indonesia, Jakarta, Indonesia; 3 Biotechnology/Intercollegiate Faculty of Biotechnology University of Gdansk and Medical University of Gdansk, Indonesia; 4 Department of Medical Pharmacy, Faculty of Medicine Universitas Indonesia, Jakarta, Indonesia; 5 Department of Animal Disease and Veterinary Health, School of Veterinary Medicine and Biomedical Sciences, IPB University, Bogor, Indonesia; 6 National Research and Innovation Agency, Cibinong, West Java, Indonesia; 7 National Veterinary Drug Assay Laboratory (NVDAL), Raya Pembangunan Gunung Sindur, Bogor, West Java, Indonesia; 8 Department of Clinical Microbiology, Faculty of Medicine, Universitas Indonesia, Jakarta, Indonesia

**Keywords:** *C. perfringens*, *in silico*, Neu5Acα-Gal, sialic acid, sialidase

## Abstract

**Objective:**

This study aims to evaluate the effect of *Clostridium perfringens* sialidase treatment on monolayer cell behavior using computational screening and an *in vitro* approach to demonstrate interaction between enzyme-based drugs and ligands in host cells.

**Materials and Methods:**

The *in silico* study was carried out by molecular docking analysis used to predict the interactions between atoms that occur, followed by genetic characterization of sialidase from a wild isolate. Sialidase, which has undergone further production and purification processes exposed to chicken embryonic fibroblast cell culture, and observations-based structural morphology of cells compared between treated cells and normal cells without treatment.

**Results:**

Based on an *in silico* study, *C. perfringens* sialidase has an excellent binding affinity with Neu5Acα (2.3) Gal ligand receptor with Gibbs energy value (∆G)—7.35 kcal/mol and Ki value of 4.11 µM. Wild *C. perfringens* isolates in this study have 99.1%–100% similarity to the plc gene, NanH, and NanI genes, while NanJ shows 93.18% similarity compared to the reference isolate from GenBank. Sialidase at 750 and 150 mU may impact the viability, cell count, and cell behavior structure of fibroblast cells by significantly increasing the empty area and perimeter of chicken embryo fibroblast (CEF) cells, while at 30 mU sialidase shows no significant difference compared with mock control.

**Conclusion:**

Sialidase-derived *C. perfringens* has the capacity to compete with viral molecules for attachment to host sialic acid based on *in silico* analysis. However, sialidase treatment has an impact on monolayer cell fibroblasts given exposure to high doses.

## Introduction

Sialidase (neuraminidase) is a large group of N-acylneuraminidase residues, the majority of which catalyze the cleavage of terminal sialic acids from complex carbohydrates on glycoproteins or glycolipids. This enzyme can be found in eukaryotes and in several pathogenic bacteria, viruses, fungi, and protozoa. Sialidase can break down sialic acid and sialo glycoprotein residues that mask or expose receptors to enzymatic interactions and ligand binding by contributing to biological functions such as cellular interactions and stabilization of glycoprotein conformations in the cell membrane [1,2]. The catalytic activity of these sialidases modulates various biological processes through conformational changes and the appearance or loss of binding sites for functional molecules. A recent study in mammals attests to the importance of the enzyme sialidase in a variety of cellular functions, including lysosome catabolism, whereas microbial sialidase appears to play a limited role in nutrition and pathogenesis [3]. Previous studies have shown that sialidase activity in vitro can be influenced by the underlying glycan composition as well as the characteristics of the mammalian sialic acid residue, such as N-acetylneuraminic acid (Neu5Ac), N-glycolylneuraminic acid (Neu5Gc), and keto-deoxy-nonulosonic acid (KDN) [4,5].

Based on the primary structure and subcellular localization, mammalian sialidases are classified into lysosomes (NEU1), cytosols (NEU2), plasma membranes (NEU3), and mitochondria (NEU4). Those sialidases are known to be particularly active against sialylated glycopeptides and oligosaccharides, have negligible activity against gangliosides, and are expressed at the highest levels in various mammalian organs [6,7]. A genetic deficiency of lysosomal sialidase results in an autosomal recessive disease associated with tissue accumulation and urinary excretion of sialylated oligosaccharides and GSLs (gangliosides) [8,9]. In contrast to mammalian sialidases, which have a complex variety of catabolic cellular functions, microbial sialidases only play a limited role in nutrition and pathogenesis [7]. Bacterial sialidase naturally plays a role in increasing its survival in the mucosal environment by utilizing nutrients from sialic acid catabolism, unmasking host cryptic ligands used for adherence, participating in biofilm formation, and modulating immune function. Bacterial sialidase is the best-studied enzyme involved in pathogenesis and can also promote commensal host association and symbiosis [10].

Based on previous studies, bacterial sialidase can potentially be used as a prevention against viral infections due to the hydrolysis of sialic acid receptors. Several sialidases that have been studied have the potential to prevent viral infections, including those derived from recombinant fusions of *Actinomyces viscosus, Pasteurella multocida,* and* Clostridium perfringens*. Bacterial sialidase can catalyze the hydrolysis of terminal sialic acid linked by *α* (2,3)-, *α* (2,6)-, or *α* (2,8) bonds to a wide variety of substrates [11–14]. In addition, some of these enzymes can catalyze the transfer of sialic acid from sialoglycans to asialoglycoconjugates by means of the transglycosylation reaction mechanism [15]. Research regarding the inhibition of viral infection by sialidase has been carried out in vitro using cell culture and in vivo using animal models [16]. However, the interactions due to sialidase administration in host cells and the impact on the behavior of these cells are poorly understood, especially since the receptors have not been independently demonstrated. Evaluation studies of novel drug development using computational methods have been used to significantly speed up the screening interaction between enzyme and ligand in the host cell with a shorter time and lower cost.

In this study, observations were made on the interaction of two types of *C. perfringens* released by sialidase NanI and NanJ in 3D crystal structure with sialic acid by molecular docking. Analysis of the genetic characterization of sialidase from wild isolates was followed by enzyme synthesis and exposure to chicken embryonic fibroblast cell culture, and observations based on the structural morphology of cells were compared between treated cells and normal cells without treatment.

## Materials and Methods

### Ethical approval

This study did not involve the use of any experimental animals; therefore, research ethics approval was not required.

### Protein choice and preparation

**Figure 1.**
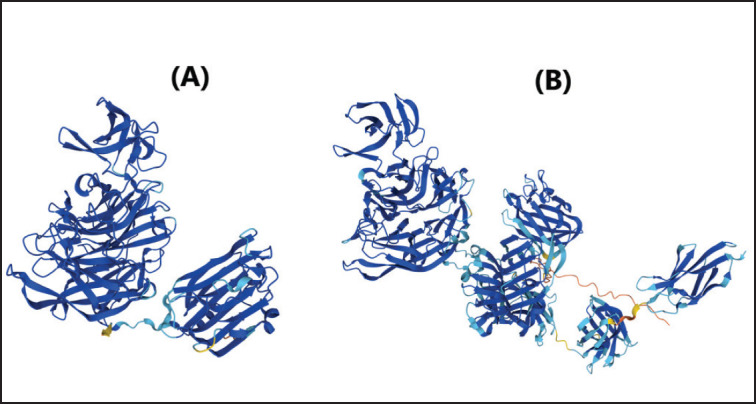
3D Structure of *C. perfringens* sialidase enzyme. (A) NanI sialidase PDB ID: AF-A0A2D0WEP9-F1. (B) NanJ PDB ID: AF-A0A2X3BTB1-F1.

The macromolecule protein utilized was the crystal structure of *C. perfringens* released sialidase, which was taken from the UniProt Knowledgebase (UniProtKB) with NanI accession No. A0A2D0WEP9 (Fig. 1A) and NanJ accession No. A0A2X3BTB1 (Fig. 1B) (http://www.uniprot.org/). Protein preparation using Auto Dock 4.2.6 software [17]. The protein is separated from the original ligand, and the water molecules are removed from the protein file, which is ready to be saved in Protein data bank (PDB) format. Polar hydrogen atoms are added, non-polar hydrogen atoms are removed, and a Kollman charge is added to the protein. Binding pockets are defined by a grid map with docking grid sizes for sialidase NanI (74 × 64 × 86) and NanJ (126 × 80 × 126).

### Preparation of ligands

A total of 2 compound ligands, sialic acid (NeuAc(a2-3) Gal and NeuAc(a2-6) Gal), were determined, and the 3D structure was searched for on the PubChem website, https://pubchem.ncbi.nlm.nih.gov/. After saving the compounds in sdf format, they were cleaned in pdb format using Open Babel. Ligands were prepared using AutoDock 4.2.6 software with files saved in pdbqt format.

### Molecular docking and interaction studies

Selected molecular docking ligands were designed with the Lamarckian genetic algorithm using Auto Dock 4.2.6 software with default parameters [18]. The output file is in dpf format; then run Autogrid4 and the Autodock4 docking process using the command prompt. The docking results were also analyzed with AutoDock 4.2.6 software to analyze residue interactions of ligand Gibbs free binding energy (Δ*G*), structural conformation, affinity, and hydrogen bonding between sialidase and ligands. Visualization of molecular docking results between ligands and proteins was carried out using Edu PyMOL and LigPlus software [17,19].

### Molecular characterization of C. perfringens sialidase genes

The presence of the sialidase gene in *C. perfringens* sialidase was carried out on isolates that had been identified as *C. perfringens* type A. Screening for the presence of the sialidase gene was carried out using the NanI and NanJ primers (Table 1). The KAPA2G Fast Hotstart Readymix polymerase chain reaction (PCR) Kit (Merck) was used in the PCR process with a total PCR reaction of 50 μl and an annealing temperature of 56°C. The sequencing of amplicons that only showed positive NanH, NanI, and NanJ of each isolate was performed by the 1st base sequencing service agency in Malaysia. Sequencing data were analyzed using MEGA X and Bioedit software to compare the nucleotide sequence of the current isolate with the sialidase gene of the *C. perfringens* isolate in the GenBank database [12,20].

### Synthesis and purification of C. perfringens sialidase

Production of native sialidase was performed under anaerobic conditions at 37°C overnight, and during the production process, the pH was maintained at 7. The medium for cultivation of *C. perfringens* type A consisted of trypticase, yeast extract, cysteine hydrochloride, and NaCl 1.0%, pH 7.4. The final culture was cooled and centrifuged to remove cells. The separated supernatant was then treated with a decrease to pH 5 to inactivate the toxin activity, and then the protein was precipitated with ammonium sulfate. Afterward, the dialyzed residue was purified by ion exchange using Q Sepharose^®^ Fast Flow (Merck, Germany), and final purification was performed using affinity chromatography with oxamic acid agarose before being kept at −20°C. Purified sialidase enzyme activity was observed using the Neuraminidase assay kit MAK121 (Sigma-Aldrich) with appropriate protocol procedures to obtain a quantitative value in U/ml [13,14].

### Chicken embryo fibroblast cell culture treatment by sialidase

Chicken embryo fibroblast (CEF) monolayer cultured cells were obtained from SPF embryonated chicken eggs (ValoBioMedia GmbH) at an age of 8–10 days. The embryo was taken and rinsed briefly with phosphate-buffered saline (PBS). Dermal skin from the dorsal region was removed with a pin set and divided into 1 mm^2^ pieces. Explants were put in a 6 cm culture dish with DMEM, 10% FBS, amphotericin (25 gm/ml), 1% penicillin, and streptomycin. Cells were grown at 37°C, 5% CO_2_, and saturated humidity, and the medium was changed every 24 h until it showed that there was monolayer CEF by microscopical examination. Monolayer CEF cells were then exposed to graded doses of sialidase ranging from 750, 150, and 30 mU. Cells were prepared in a culture maintenance medium mixture that contained 1% heat-inactivated FCS, 2% L-glutamine, 2% sodium bicarbonate, and 1% antibiotic solution (penicillin, neomycin, and streptomycin). Sialidase was added to monolayer CEF cells in a 96-well plate and incubated in a CO_2_ incubator at 37°C for 2 h. Sialidase was removed, replaced with a maintenance medium, and then re-incubated in a CO_2_ incubator at 37°C for 24 h. This procedure is in accordance with previous studies that observed the effectiveness of sialidase to inhibit viral replication. Further observations were made in addition to microscopic observations of cell structure behavior as an indicator of cell response [14].

**Table 1.** Characterization of *C. perfringens* Genes [21,22].

Bakteri	Genes	Sekuen basa	Amplikon (*base pair*)	Suhu *annealing* (°C)
*C. perfringens*	*cpα* (alpha)	(F) 5′-GCTAATGTTACTGCCGTTGA-3′	324	53
		(R) 5′-CCTCTGATACATCGTGTAAG-3′		
	*nanH*	(F) 5′- CTGCAATTCAAGGTGTTGGTG -3′	285	56
		(R) 5′- CTTGTCTTCTAAGCTCATATCC -3′		
	*nanI*	(F) 5′- CAAGAGTTGGTTTTGAGC -3′	467	56
		(R) 5′- AAATAAGGCTGGTATTCTG -3′		
	*nanJ*	(F) 5′- AATTGGATGGCTAGGTGGAGTT -3′	306	56
		(R) 5′- CAGGTGCTTCCTAAATCGTGAG -3′		

### Cell behavior analysis

Analysis of cell viability CEF monolayers were examined based on cell death that lost their adherence by crystal violet staining in a culture. This protocol describes a quick and reliable screening method that is suitable for the examination of the impact of foreign compounds on cell survival and growth inhibition. The viability of the cell is determined by the percentage of treated cells that are viable (attached) by comparing the average OD 570 values of stimulated cells with the OD 570 values of the non-treated cells that are set to average 100% viability [23,24]. Cell structure was analyzed and quantified using the ImageJ software. Microscopy images were used to calculate cell count, total area, perimeter, and solidity. Cell shape is represented by a set of lines located at the midpoints of the boundaries of individual cells, resulting in a skeletal representation of the overall shape that can be used to reconstruct the entire shape.

### Statistical analysis

The data were analyzed in Graphpad Prism 9.1.2. The mean and standard error of the mean, or SD, were used to display normally distributed data. The data were analyzed by one-way ANOVA and post hoc least significant difference to determine the significance of differences among the treatment groups. *p* ≤ 0.05 was considered statistically significant.

## Results 

### In silico docking simulation of C. perfringens sialidase with Neu5Acα (2–6) and Neu5Acα (2–3) ligands

**Figure 2.**
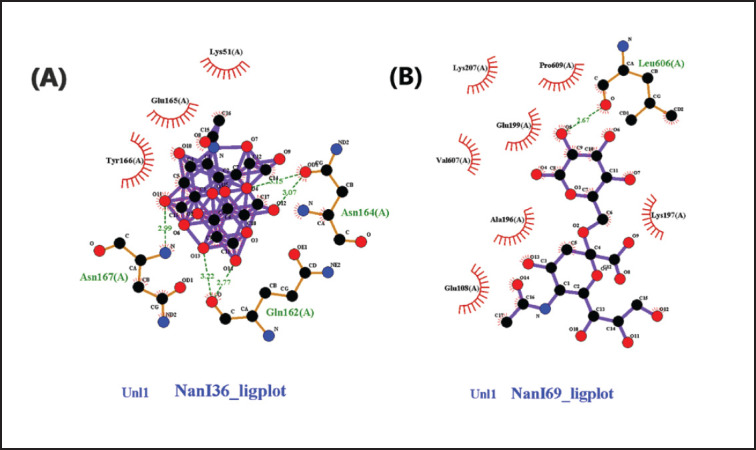
Interactions of NanI sialidase enzyme in 2D with sialic acid sialic acid Neu5Acα (2–3) Gal (A) and Neu5Acα (2–6) Gal (B). Hydrophobic interactions are depicted as half-moons, whereas dotted green lines with distances in angstrom represent hydrogen bonding.

**Figure 3.**
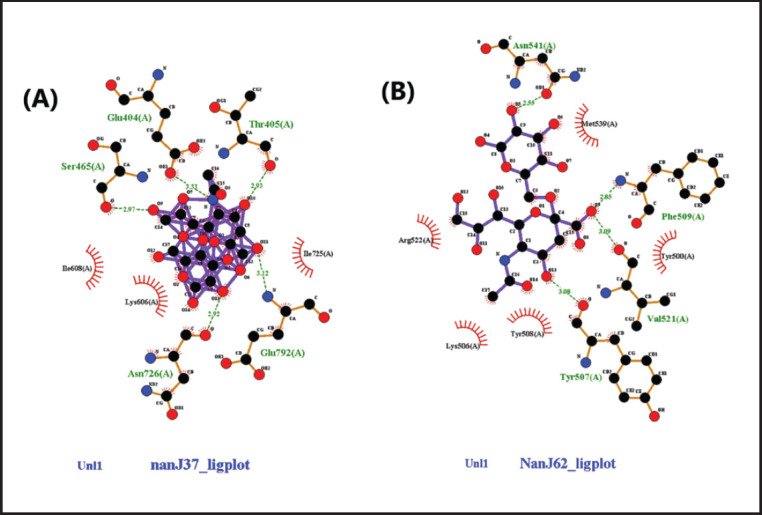
Interactions of NanJ sialidase enzyme in 2D with sialic acid sialic acid Neu5Acα (2–3) Gal (A) and Neu5Acα (2–6) Gal (B). Hydrophobic interactions are depicted as half-moons, whereas dotted green lines with distances in angstrom represent hydrogen bonding.

Based on the result of docking analysis between sialidase macromolecule and sialic acid ligand, it shows that interaction between NanJ and sialic acid NeuAc(a2-3) Gal has the lowest bond energy with the Gibbs free energy or ∆G of − 7.35 kcal/mol and the inhibition constant (Ki) was 4.11 µM compared with NeuAc(a2-6) Gal (Table 2). According to the interaction study, NeuAc (a2-3) forms five hydrogen bonds at the binding site of the NanJ sialidase enzyme. This hydrogen bond of 2.92, 2.93, 2.97, 3.12, and 3.33 Å length was found in Asn 726, Thr 405, Ser 465, Glu 792, and Glu 404, respectively, with the –OH group of ligands (Table 3). The interaction profile of NeuAc(a2-3) and NanI ligand was quite interesting, as shown in Figure 2, with three hydrogen bonds, two of which are forms with Gln 162 (2.77 and 3.22 Å) and Asn 164 (3.07 and 3.15 Å). Likewise, three hydrophobic interactions were also detected with surrounding residues (Lys 51, Glu 165, and Tyr 166). Additionally, the surrounding residues Ile 608, Lys 606, and Ile 725 were examined for the three hydrophobic interactions (Fig. 3).

Based on the results of the docking simulation of the sialic acid receptors, *C. perfringens* sialidase was found to have a higher binding interaction propensity with the sialic acid Neu5Acα (2–3) receptor based on the lowest bond energy values. Tables 2 and 3 show the conformation of NanI and NanJ with bond energy values of -4.63 and -7.35 kcal/mol at the inhibition constants (Ki) of 404.57 and 4.11 µM, respectively.

### Clostridium perfringens sialidase genes

**Figure 4.**
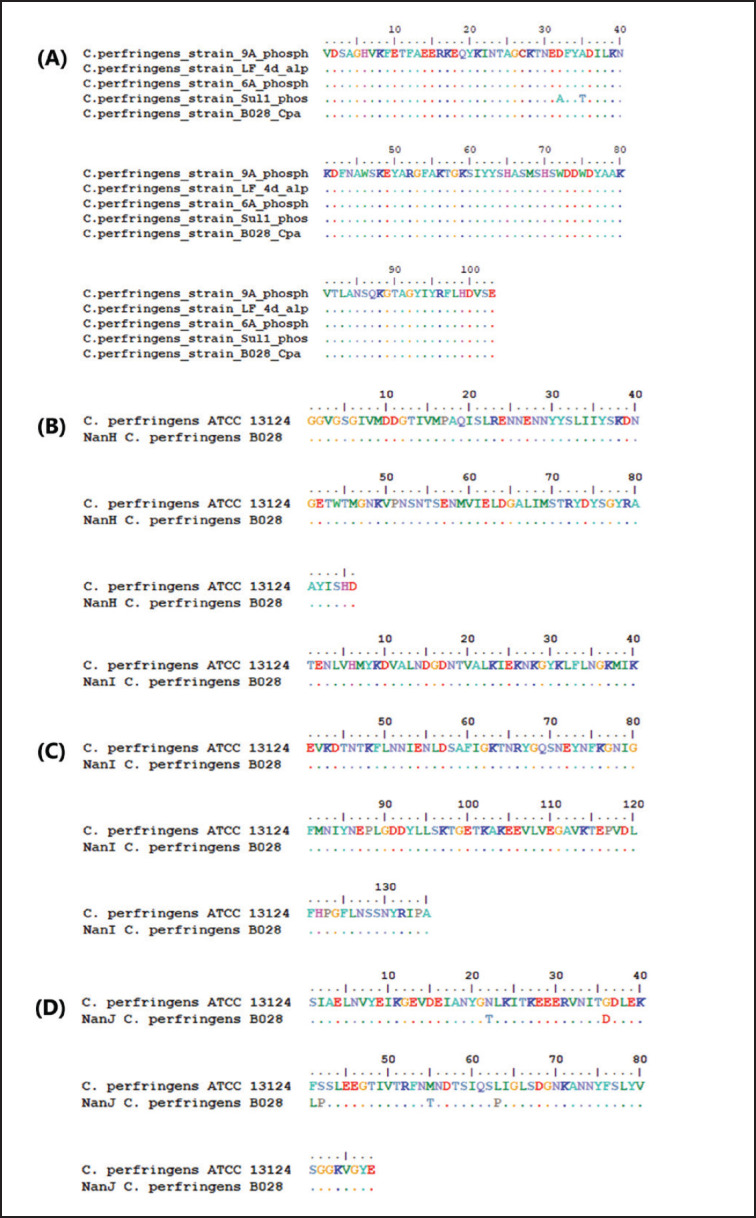
Toxin and sialidase sequences of *C. perfringens* B028 amino acids. (A) Nucleotide sequencing results of alpha toxin gene (plc) amino acid translation results compared to isolates in Genbank. (B–D) Sequencing analysis of amino acid (NanH, NanI, and NanJ) sialidase compared to *C. perfringens* ATCC 13124 from Genbank.

Molecular characterization by PCR of the gene encoding sialidase was carried out on selected *C. pefringens* type A isolates that contain three sialidase genes that have been used in previous studies. Based on this result, the sialidase gene amplicon was confirmed by the presence of a band of PCR electrophoresis at 285 bp for NanH, 467 bp for NanI, and 306 bp for NanJ sialidase. Sequence analysis of nucleotides was compared to several reference strain plc genes from Genbank and showed that isolates of *C. perfringens* type A in this study had a similarity of 99.1%–100% at amino acid position 103 (residues 171–273) of 398 aa (Table 4). Meanwhile, results of sequencing sialidase genes NanH and NanI at positions of amino acid residues 174–259 from 382 aa and residues 94–228 from 653 aa found a 100% similarity. Analysis of NanJ showed different results compared to the reference isolate, with a similarity of 93.18% at the position of 172–259 amino acid residue from 1173 aa (Fig. 4).

**Table 2.** Molecular docking details of sialic acid receptors against *C. perfringens* NanI sialidase enzyme.

Ligand structure	Hydrogen bonding residue and distance		Hydrophobic interacting residues	Gibbs energy (Δ*G*)	Inhibition constant (Ki)
α (2,3)-Gal	Gln 162 Asn 164 Asn 167	2.77 Å 3.22 Å 3.07 Å 3.15 Å 2.99 Å	Lys 51 Glu 165 Tyr 166	−4.63	404.57 µM
α (2,6)-Gal	Leu 606	2.67 Å	Lys 207 Val 607 Pro 609 Glu 199 Ala 196 Glu 108 Lys 197	−2.59	12.7 mM

**Table 3.** Molecular docking details of sialic acid receptors against *C. perfringens* NanJ sialidase enzyme.

Ligand structure	Hydrogen bonding residue and distance		Hydrophobic interacting residues	Gibbs energy (Δ*G*)	Inhibition constant (Ki)
α (2,3)-Gal	Asn 726 Thr 405 Ser 465 Glu 792 Glu 404	2.92 Å 2.93 Å 2.97 Å 3.12 Å 3.33 Å	Ile 608 Lys 606 Ile 725	−7.35	4.11 µM
α (2,6)-Gal	Asn 541 Phe 509 Tyr 507 Val 521	2.55 Å 2.85 Å 3.08 Å 3.09 Å	Arg 522 Lys 506 Tyr 508 Met 539 Tyr 500	−3.51	2.68 mM

### Synthesis and purification of C. perfringens sialidase

The result of the synthesis of native sialidase production derived from *C. perfringens* supernatant culture is known as crude sialidase, with a total protein concentration of 0.135 mg/ml and a specific activity of 0.44 U/mg. Initial purification with 70% saturated ammonium sulfate causes an increase in protein concentration to 4.1 mg/ml with a specific activity of 0.5 U/mg. The next step was purification using ion exchange so that the specific activity increased to 10.25 U/mg, followed by a decrease in protein concentration to 0.4 mg/ml. The last purification by affinity chromatography using oxamic acid agarose also led to a drastic increase in activity against the sialidase enzyme to 75 U/mg (Table 5). The purified sialidase is then stored at -20°C and can be used for further treatment tests on cultured cells to observe the structure of cell behavior.

### Cell culture response to sialidase

**Figure 5.**
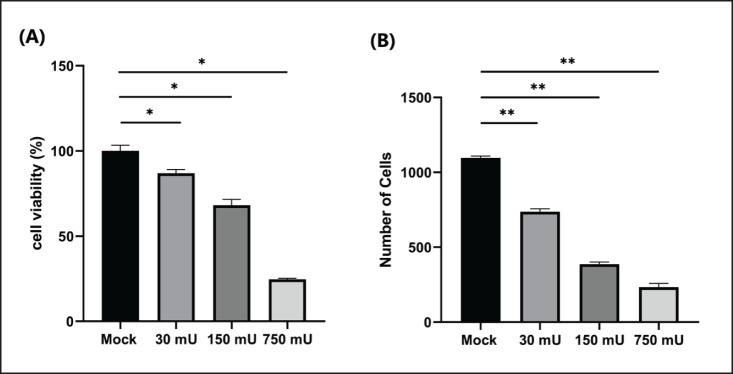
Viability and number of treated cells. (A) Cell viability analysis was measured to determine the toxicity of sialidase based on cell attachment to the plate with crystal violet staining. (B) Visual cell counting analysis using ImageJ.

**Figure 6.**
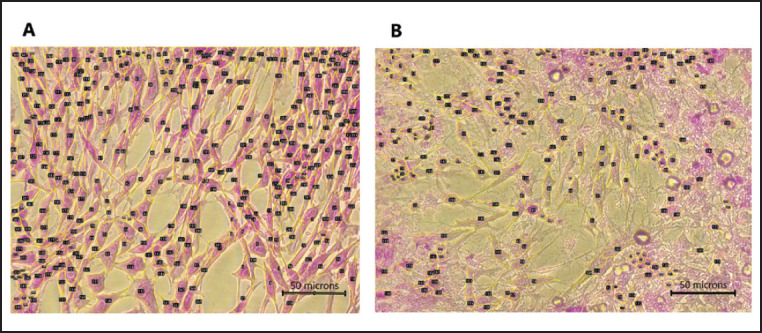
Cell count and structure analysis of treated CEF cells. Measurement of the cells was carried out by detecting the boundaries and distances between cells based on the visible structure of the cytoplasm and nucleus of fibroblast cells. (A) Cells treated with sialidase 150 mU, whereas (B) treated with sialidase 750 mU.

Observation of CEF cells against sialidase administration at doses of 750, 150, and 30 mU was carried out based on the proportion of CEF cells attached to microplates by crystal violet staining, which showed that there were significant differences between each dose. Administration of sialidase at the highest dose caused a decrease in remaining cell viability until it reached 24.7% ± 0.55, while the middle and lowest doses were 68.15% ± 3.44 and 86.98% ± 2.13, respectively (Fig. 5). Absorbance data is calculated by the following formula: Viability cell percentage (%) = [(Absorbance of treatment cells − Absorbance background)/(Absorbance control cells − Absorbance background)] × 100%. This was also reinforced by an analysis of the number of cells that were observed microscopically, which was then analyzed based on the shape of the structure and then counted (Fig. 6). The results show a correlation between the analysis of cell viability and optical observations of the number of cells. Administration of sialidase with the highest dose caused a drastic decrease in cells compared to the group without treatment, and so on, which shows that the higher dose of sialidase given will cause a significant decrease in cell numbers.

**Table 4.** Pairwise Comparison of *plc* gene *Clostridium perfringens* type A B028.

No.	*GenBank* Accession *Number*	*C. perfringens* strains	1	2	3	4	5
			KT020603	JQ071567	KT020600	MN224676	
1	KT020603	strain_9A_phospholipase_C					
2	JQ071567	strain_LF_4d_alpha_toxin_gene	100				
3	KT020600	strain_6A_phospholipase_C	100	100			
4	MN224676	strain_Sul1_phospholipase_C	99.1	99.1	99.1		
5		B028	100	100	100	99.1	

**Figure 7.**
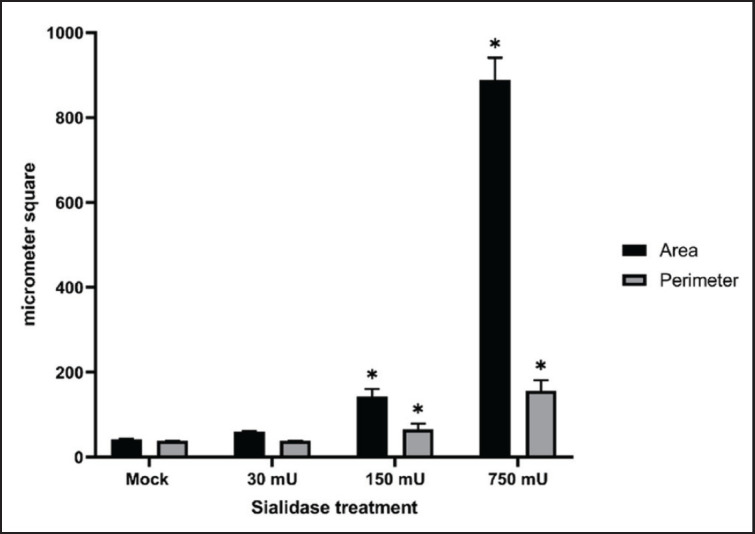
Analysis of area and perimeter compared between normal and treated CEF cell. Measurements of the area and perimeter show that there appears to be a drastic increase in the administration of high doses of sialidase which results in changes in the structure of cells.

**Figure 8.**
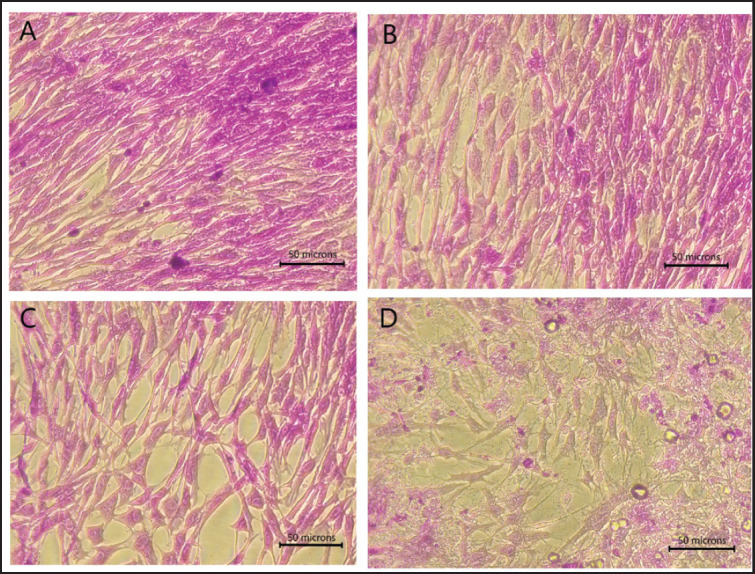
CEF cell crystal violet staining of treated cells at 400× magnification. Observations on cells treated with several sialidase doses showed significant structural changes, especially in the distance between cells and the cell surface compared to normal cells without treatment. (A) Normal CEF cells without treatment. (B) Cell treated with 30 mU sialidase. (C) Cell treated with 150 mU sialidase. (D) Cell treated with 750 mU sialidase.

The observation of changes in cell behavior based on cell structure was based on area and perimeter microscopic images as measured parameters. The results showed that the cells treated with 750 and 150 mU of sialidase significantly increased the empty area and perimeter of CEF cells compared with mock control cells (Fig. 7). This increase is accompanied by a difference in the microscopic images compared to normal groups that appear to fill the surface. At the highest dose of sialidase, it seems that more empty areas with fragmented cell structures appeared (Fig. 8).

## Discussion

The interaction between sialidase NanI and NanJ with sialic acid receptors has been successfully illustrated, predicted, and analyzed in this study using in-silico analysis of a 3D model of the sialidase enzyme from *C. perfringens* obtained from the PDB data bank. The potency and evaluation of sialidase were known to have the ability to inhibit viral infection both in ovo and *in vitro*; this was supported by a decrease in the ability of viral replication in exposed host cells [11–13]. This study assured and encouraged this discovery, as shown by the ability of sialidase, which has the lowest Gibbs energy value (ΔG) –7.35 with a Ki value of 4.11 µM, towards sialic acid α(2.3)-Gal receptors. This demonstrates its capacity to outcompete viral molecule competitors and attach to sialic acid receptors. In this instance, the bond energy is the free energy (Δ*G*) associated with the level of spontaneity of a reaction. This reaction runs if the Gibbs energy value is less than 0 or a negative value, which indicates a spontaneous formation of the protein-ligand complex due to the stability and complex strength of the noncovalent interactions. Related to Gibbs energy value (Δ*G*), Ki can be used to approximate the ability of a compound to inhibit the interaction between virus molecules and receptors (ligands).

**Table 5.** Purification step of *C. perfringens* sialidase.

Purification step	Volume (ml)	Total protein (mg)	Protein recovery	Activity (U/ml)	Total activity (U)	Recovery activity	Spesific activity (U/mg)	Purification fold
Crude sialidase	1000	135	100%	0.06	60	100%	0,444	1
Ammonium sulphate	20	84	62%	2.1	42	70%	0,500	1.14
Ion Exchange	10	4	3%	4.1	41	68%	10,250	23.35
Affinity Chromatography	15	0,3	0.2%	1.5	22.5	38%	75,000	170.83

The values of ∆*G* and Ki determine the affinity; the lower these values, the higher the affinity of the docked ligand and the more stable the bond that occurs [12,25]. In addition, hydrogen bonding parameters are also the most important specific interaction in the process of interaction between ligands and receptors. In this study, the hydrogen bonding interaction of *C. perfringens* sialidase with *α* (2.6)-Gal was shown to be overall below 3.3 Å, while *α* (2.3)-Gal was overall below 3.4 Å. This contributes to the increased molecule affinity for the target protein, which forms an electrostatic interaction between the hydrogen donor and acceptor. In a hydrogen bond interaction analysis, the criteria for hydrogen bonding require a hydrogen donor and acceptor with a bond distance of less than 3.9 Å [26–28].

The molecular characterization of sialidase used in this study was carried out to confirm and analyze the similarity of the isolate by three primer pairs (NanH, NanI, and NanJ) with an additional plc gene that is generally used to identify the *C. perfringens* toxinotyping gene [29]. 29 Based on the result, NanH and NanI sialidase *C. perfringens* isolates in this study had 100% similarity of sialidase amino acids compared with the reference strain ATCC 13124 found in GenBank, while NanJ had 93.18% similarity. This finding revealed that NanJ has the most variation in similarity with NanI or NanH among *C. perfringens* reference isolates and may impact different expressions of sialidase for their tendency to hydrolyze sialic acid [30,31].

Bacterial sialidase was obtained by eliminating other substances originating from the crude supernatant of *C. perfringens* culture, considering the many other substances that are also produced by the bacteria. The results of sialidase purification in this study were known to be effective in reducing other substances derived from the supernatant based on a decrease in the total amount of protein and an increase in specific activity after ion exchange and affinity chromatography purification steps. The final step of purification using oxamic agarose in affinity chromatography efficiently purifies specific sialidase enzymes based on increased sialidase activity [32].

Administration of purified sialidase in this study appears to have an impact on the cell structure of chicken embryonic fibroblasts. The higher the concentration of sialidase given, the more it seems that there is a very visible change in the structure and properties of the cell. *Clostridium perfringens* sialidase may cause a decrease in cell monolayer viability at high doses, causing cells to appear to lose cell-cell contacts, resulting in rather evenly dispersed individual cells, and impacting changes in the perimeter of the cell. It demonstrates that the removal of sialic acid from the cell surface due to sialidase is able to cause slight changes in the pattern of glycosylation, which are known to cause dramatic changes in cellular behavior [14,^33^,34]. In this study, the appearance of the CEF cells was characterized by an increase in the empty space area between cells, which was most likely due to the loss of sialic acid on the cell surface. On the other hand, compared to the control group, administration of sialidase at a low dose of 30 mU did not cause a significant increase in perimeter and area or changes in cell structure. However, further in-vivo studies using animal models need to be carried out to find out the side effects of sialidase treatment, notably how variations in the cell structure may affect the different results that are needed to test the fundamental safety of novel drugs.

## Conclusion

*In silico* analysis based on Gibbs energy value (Δ*G*), Ki, and hydrogen bonds between the sialidase enzyme of *C. pefringens* sialidase and the Neu5Acα (2-3/2-6) Gal ligand supports the notion that they have excellent binding affinity. Bacterial sialidase derived from *C. perfringens* had a significant impact on the properties of monolayer CEF cells exposed, depending on the dose administered. These results provide basic information regarding the impact of the administration of purified sialidase from wild isolates on the morphological structure of CEF monolayer cells.

## List of abbreviations

AA, amino acid; Asn, Asparagine; ANOVA, Analysis of variance; Å, Ångström; CEF, Chicken embryo fibroblast; Gln, Glutamine; h, hour; Lys, lysine; ml, milliliter; OD, Optical density; PDB, Protein data bank; PCR, Polymerase chain reaction; SDF, Spatial data file; SD, Standard deviation; U, Unit; Tyr, Tyrosine.
